# Are secondary cities in Asia and Africa ready for food systems transformation? Policy insights from Bangladesh, Kenya, Rwanda, and the Philippines

**DOI:** 10.3389/fpubh.2026.1852336

**Published:** 2026-08-31

**Authors:** Daniel Gacutan Salunga, Kaspar Wyss

**Affiliations:** 1 Swiss Tropical and Public Health Institute, Allschwil, Switzerland; 2 University of Basel, Basel, Switzerland

**Keywords:** content analysis, food systems, nutrition, policy mapping, secondary cities

## Abstract

**Background:**

Policies are essential tools for food systems transformation to reshape production, distribution, and consumption; enabling sustainable, healthy, and equitable outcomes and simultaneously address malnutrition, climate change, and social inequalities. This systematic policy mapping and content analysis aimed to synthesize available nutrition and food systems policies and action plans in secondary cities in Bangladesh, Kenya, Rwanda, and the Philippines, describing characteristics, types, thematic and food value chain focus, and food security and nutrition outcomes.

**Methods:**

In 2024 and 2025 documents were systematically collected across eight cities in four countries through formal and informal channels. 89 documents dated from January 2015 to 2025 were assembled and underwent eight steps of systematic policy mapping and content analysis following the food systems framework.

**Results:**

Thirty-six screened documents met eligibility criteria and were included in the analysis. Nutrition governance and interventions are the focus of most policies. Cities differ in priorities. Gaps exist on the food value chain relating to storage, transport and trade, and processing and transformation. For outcomes, healthy diets emerged as the primary area of focus while food stability is the least addressed. Limited measures to manage shocks and seasonal volatility were seen.

**Conclusion:**

Addressing policy gaps in the food value chain and improving measures for food stability is essential. Strengthening policy foundation through multisectoral engagement and evidence-based governance is key. Future studies should examine implementation of key policies and action plans at the local level.

## 1 Introduction

Food systems transformation refers to the systemic reconfiguration of how food is produced, traded, governed, and consumed to achieve sustainable, healthy, and equitable outcomes (1). Nutrition and food systems policies are among the essential inputs that can drive and govern this transformation because food systems are significant drivers of undernutrition, obesity and climate change, well-designed policies can address more than one of these crises simultaneously (2–4). They can shape food prices, availability, marketing, composition and labeling, making healthier options the default rather than an individual choice (2, 5, 6). Beyond influencing consumer food environments, food systems regulation and policy action can also help align agriculture, health, trade, climate adaptation, and environmental objectives. Increasingly, national agriculture and food policies are being linked to broader global commitments including the Sustainable Development Goals (SDGs) and Nationally Determined Contributions under the Paris Agreement, particularly those related to SDG (2) Zero Hunger, (3) Good Health and Well-being, (11) Sustainable Cities and Communities, and (13) Climate Action (7). For example, aligning agricultural, trade, social protection, and urban planning policies with nutrition goals can shift food systems from a focus on calories alone toward healthy nutrition and sustainability (3, 4, 8, 9). Well-designed policies, such as school meal programs and targeted subsidies, can also reduce socio-economic and Indigenous nutrition inequities (2, 10, 11).

Low- and middle-income countries (LMICs) are experiencing rapid urbanization, with population movements increasingly directed not only toward megacities but also toward secondary cities that serve as emerging economic hubs. According to the United Nations World Urbanization Prospects 2018, nearly 90% of global urban population growth will occur in Africa and Asia, with secondary cities absorbing much of this expansion (12). This demographic transition is reshaping the geography of malnutrition, as increasing urban populations face the simultaneous burdens of undernutrition, micronutrient deficiencies, overweight and obesity, and diet-related non-communicable diseases (NCDs) (13).

Secondary cities such as the sites of interest to this study (Dinajpur and Rangpur, Bangladesh; Bungoma and Busia, Kenya; Rubavu and Rusizi, Rwanda; and Baguio and Cauayan, Philippines) are typically smaller, non-primary urban centers that function as intermediary hubs linking rural food production areas with urban consumers. In food systems terms, these cities play important roles in agricultural trade, food distribution, informal food markets, labor mobility, and regional supply chains, while simultaneously experiencing rapid dietary and food environment transitions. Despite their strategic role in connecting rural and urban food systems, secondary cities remain under-researched sites for food systems governance and nutrition policy implementation (14, 15). Unlike capital cities, they often operate with limited institutional capacity, reduced policy visibility, and complex decentralization arrangements that challenge policy implementation and coordination (16, 17). Understanding how these cities govern food and nutrition issues is therefore essential for addressing emerging urban health inequalities.

Improving urban diets requires comprehensive, multi-sectoral food system governance that spans from production to consumption and encompasses agriculture, markets, social protection, environmental management, and animal and human health (18–20). Because urban centers consume approximately 70% of global food, their local policies and food availability strongly dictate broader health outcomes (21–23). Within this landscape, rapidly transitioning secondary cities are particularly important, acting as hubs that connect rural and urban food systems through regional markets and shorter, sustainable supply chains (24–27). Despite this importance and the reality that secondary cities in countries like Bangladesh, Kenya, Rwanda, and Philippines face food insecurity and double burden of malnutrition, including undernutrition, micronutrient deficiencies, overweight, and obesity (24, 25, 28, 29) they remain underrepresented in global food policy research. Most existing literature focuses on high-income cities, leaving significant knowledge gaps regarding how food systems are governed and policies are implemented in LMIC secondary cities that have vastly different institutional capacities (30, 31).

Despite growing attention to the role of cities in shaping food systems, secondary cities face persistent governance challenges. Existing research highlights fragmented policy landscapes in LMIC urban settings, where responsibilities for nutrition, agriculture, markets, and social protection are distributed across multiple agencies with limited coordination (32). While nutrition specific interventions such as supplementation, feeding programs, and maternal child health services are frequently present, city-level policies and regulatory frameworks often lack a comprehensive food systems perspective, with limited attention to food processing standards, retail regulation, market governance, informal food vendors, food safety oversight, or unhealthy food marketing practices that shape urban food environments in secondary cities (33, 34).

This study applies a food systems framework based on the model developed by Van Berkum et al. (35) and adapted by Borman et al. (36). The framework uses systems thinking to analyze interactions across the food supply value chain, including agricultural production; storage, transport and trade; processing and transformation; retail and provisioning; and food consumption, together with their environmental, social, political, and institutional drivers. The framework also categorizes food security and nutrition outcomes into five interconnected domains: food availability, access, stability, food use, and healthy diets. In this study, the framework guided the mapping and comparative analysis of nutrition and food systems policies across the selected secondary cities. By analyzing these complex feedback loops and outcomes, policymakers can design strategic interventions aimed at achieving sustainable nutrition, equitable economic development, and environmental health (36, 37).

Evidence indicates that multi-component, systemic, city-led policies such as procurement, planning, regulation, education, social protection can improve diet quality and equity, especially when aligned across scales and supported by city networks, a good example for this is the Milan Urban Food Policy Pact (2, 22, 38, 39). Nutrition and food system policies in cities, particularly in secondary cities that bridge rural production and urban demand, are central to tackling malnutrition and building resilient, sustainable food systems. Evidence supports integrated, city led, multisector policies, but rigorous, context specific evaluation in secondary cities remains a research priority.

Secondary cities in LMICs are undergoing rapid demographic, economic, and food environment transitions that intensify pre-existing inequities and create new risks for urban health (40, 41). These transitions underscore the need for evidence to inform governance reforms and assess policy readiness for healthier, more sustainable food systems. In response, this study systematically maps and compares nutrition and food system policies and plans from secondary cities in Bangladesh, Kenya, Rwanda, and the Philippines. Using the food systems framework, the study examines policy types, governance domains, food value chain coverage, and food security and nutrition outcome areas to generate insights into the readiness of local governments for food systems transformation. The findings provide evidence to support multi-sectoral and multilevel governance reforms aimed at strengthening urban food systems, improving nutrition outcomes, and advancing sustainability and climate resilience objectives in rapidly growing secondary cities. This study is part of the NutriPoliCities Study on Nutrition and Food Systems policies and action plans, and food environment types and consumer perceptions in Secondary Cities in Bangladesh, Kenya, Rwanda and the Philippines linked with the Nutrition in City Ecosystems (NICE) Project (25) at the Swiss Tropical and Public Health Institute.

## 2 Methods

### 2.1 Study setting

This study focuses on eight secondary cities across four countries: Dinajpur and Rangpur in Bangladesh; Bungoma and Busia in Kenya; Rubavu and Rusizi in Rwanda; and Baguio and Cauayan in the Philippines. The selection comprises six cities from the NICE project and two additional cities in the Philippines. The inclusion of the Philippines as a country site was aligned with the initially approved research funding from the Swiss Government Excellence Scholarship Program. Within the Philippines, Baguio City and Cauayan City were selected based on the following criteria of comparable population sizes and urban growth patterns and facing similar nutrition and food systems challenges. These study sites represent diverse nutritional landscapes see Table 1, particularly regarding stunting prevalence in children under five. Stunting rates are notably high in the Rwandan cities of Rubavu (35%) and Rusizi (21%), followed by the Philippine cities of Cauayan (29% at the provincial level data, since no specific national survey data for Cauayan City) and Baguio (23%) which were above 20% and considered of high public health significance (42). In Bangladesh, stunting affects 22% of children in Rangpur and 16% in Dinajpur, while the Kenyan cities show the lowest rates in this group at 13% in Bungoma and 8% in Busia.

**Table 1:** Demographic profile and status of malnutrition in children under 5 years in the study sites.

City	Population size	Total surface area	Stunting under 5 children
Dinajpur (BD)	300,000 (2021)	25 km^2^	16%
Rangpur (BD)	800,000 (2021)	206 km^2^	22%
Bungoma (KE)	270,000 (2019)	56 km^2^ (County)	13%
Busia (KE)	110,000 (2019)	45 km^2^	8%
Rubavu (RW)	150,000 (2018)	45 km^2^	35%
Rusizi (RW)	70,000 (2019)	88 km^2^	21%
Baguio (PH)	366,358 (2020)	57.51 km^2^	23%
Cauayan (PH)	43,403 (2020)	336.40 km^2^	29% (Isabela province)

BD, Bangladesh; KE, Kenya; RW, Rwanda; PH, Philippines. Data source: (1) Stunting data of NICE cities in BD and KE—Barth-Jaeggi et al. (24), RW cities—Ntawuyirushintege et al. (80), PH cities—2018 Expanded National Nutrition Survey (28, 29).

The governance frameworks for addressing these nutrition outcomes vary significantly by national context (see Supplementary material Section 1, Table 1). Bangladesh and Rwanda utilize unitary systems where policy is set centrally by bodies such as the Bangladesh National Nutrition Council and the Rwandan Ministry of Health, though implementation is routed through decentralized health tiers or district structures. Conversely, Kenya and the Philippines operate under more devolved structures. Kenya’s constitutional devolution empowers counties to implement specific County Nutrition Action Plans, while the Philippines’ Local Government Code mandates that Local Government Units (LGUs) operationalize the Philippine Plan of Action for Nutrition (PPAN) with national oversight.

### 2.2 Data collection and study design

This study employs policy mapping, a systematic research method that adapts the principles of content analysis to the evaluation of proposed or enacted legal frameworks. The content analysis was guided by Drisko and Maschi (43); and Krippendorff (44). While frequently applied to federal and state legislation, the utility of policy mapping extends to diverse regulatory formats, including local ordinances and administrative regulations established by Burris et al. (45). Policy mapping follows a defined set of eight steps which underlined the present analysis: (1) identify a research question, (2) identify a data source, (3) specify policy inclusion and exclusion criteria, (4) develop categories and processes for coding, (5) build a data set, (6) code policies, (7) analyze coded data, and (8) synthesize and disseminate results (45).

In Step 1 (Identify a research question), the investigation focused on the specific characteristics of existing policies and action plans within selected cities. Step 2 (Identify a data source) involved a dual-channel collection strategy: formal channel through request letters and meetings with city governments and project coordinators, and informal channel through retrieval via national and city databases, council archives, and stakeholder networks. The policies and action plan documents were collected in the last quarter of 2024 to the first quarter of 2025. For Step 3 (Specify policy inclusion and exclusion criteria) see Table 2, documents were evaluated based on their origin, relevance to food systems (e.g., health, agriculture, safety), and temporal relevance between January 2015 and January 2025. Exclusion criteria filtered out private-sector documents, academic papers, and general administrative reports lacking substantive food or nutrition content.

**Table 2:** Inclusion and exclusion criteria for nutrition and food systems policies and plans.

Inclusion criteria: a document is included if it
Is issued by the city government or statutory bodies and is accessible, and readable
Is drafted, enacted and implemented nutrition and food system policies and action plans
Relates to nutrition, food systems, food security, health, agriculture, markets, food safety, or consumer environments
Covers or affects the selected secondary cities
Falls within the study period (January 2015 to January 2025, most recent version of the policy or plan was used)
It is in English or in local language—if in local language, it will be forwarded to research partners for translation however all policies and plans collected are in English

Exclusion criteria: exclude documents that
Are general development plans without any food or nutrition content
Are private-sector documents
Are academic research papers
Have no relevance to food, nutrition, or food systems
Are program, activity and administrative reports documents in nature
Are past versions of the policy or plans

In Step 4 (Develop categories and processes for coding), a comprehensive table of operational definitions and a coding guide were created to ensure analytical consistency (see section 2.3 for detailed coding process). Step 5 (Build a data set) involved the creation of a grid content matrix in Microsoft Excel, capturing variables such as policy objectives, policy types adapted based on the definitions by Dwyer (46), policy focus adapted from Adhikari et al. (47), value chain positioning, and nutrition and food security outcomes based on the food system framework by Borman et al. (36) adapted from Van Berkum et al. (35); this stage included a preparatory data cleaning phase to standardize document layouts and enhance readability.

During Step 6 (Code policies), all documents underwent an initial content scan and screening, followed by two rounds of review to verify their inclusion status. A basic screening was conducted for all included policy documents prior to analysis. This screening assessed readability (clarity and comprehensibility of the policy text), and language accessibility (all 36 policies were available in English; policies from Bangladesh were originally in Bengali, and English-translated versions were provided by project partners). The provided English-translated versions of the nutrition action plans in Bengali were verified by Bangladeshi project partners. A complementary quality appraisal using a more detailed evaluative framework, reflecting the methodological and conceptual depth required for this assessment, will be presented in a complementary manuscript.

In Step 7 (Analyze coded data) consisted of two rounds of content analysis and criteria rating. Discrepancies between the first two rounds were resolved by following the second analysis, unless differences exceeded 20%, which triggered a definitive third analysis. Finally, in Step 8 (Synthesize and disseminate results), data were synthesized through frequency distributions and heat maps to generate narrative insights for dissemination via stakeholder presentations and peer-reviewed publication. See Supplementary material Section 2 for the code book that consists of operational definition of terms and Section 3 for the list of policy and action plan documents.

### 2.3 Data analysis: coding process

All included policy and action plan documents were coded using a codebook developed for this study and informed by the food systems framework (35, 36). The unit of analysis was the individual policy or action plan document. Coding assessed four main analytical dimensions: (1) policy type classification, (2) thematic focus, (3) food supply system value chain focus, and (4) food security and nutrition outcomes. Each document underwent full text review and was coded using predefined operational definitions and decision rules (see Supplementary material for the complete definitions). Policies were first classified into one mutually exclusive category: Nutrition Policy, Food Policy, Food and Nutrition Policy, or Food and Nutrition Action Plan. Classification was based on the document’s primary purpose, scope, and implementation characteristics.

Following classification, documents were assessed across thematic domains, food value chain domains, and food security and nutrition outcome domains using a three-point ordinal scoring system (Green = Full, Yellow = Partial, Red = None). Each domain was coded independently, allowing a single document to receive scores across multiple categories. The following criteria were hereby used: Full Presence (Green): The domain was a central focus of the policy or action plan and was explicitly identified in strategic sections (e.g., mission, goals, objectives, or contextual framing) and was also reflected in implementation components such as activities, outputs, indicators, strategies, or operational actions. A domain was coded as Full Presence (Green) when both strategic-level and operational level evidence were covered. For example, a policy that explicitly established multi-sectoral nutrition coordination committees, defined institutional responsibilities across health, agriculture, and local government sectors, and included budgeting, accountability, and implementation mechanisms for nutrition actions was coded as having full presence for the Nutrition Governance domain. Partial Presence (Yellow): The domain was present but not consistently or not a central part of the policy. These included domains mentioned only in strategic sections (e.g., mission, objectives, or situational analysis) without corresponding implementation actions, or domains reflected in activities or outputs without being identified as a strategic priority. A domain was coded as Partial Presence (Yellow) when the issue was acknowledged but incompletely operationalized. For example, a policy that mentioned inter-sectoral coordination or assigned nutrition-related responsibilities to government agencies, but lacked clear operational structures, accountability mechanisms, or implementation strategies, was coded as partial presence for the Nutrition Governance domain. None (Red): The domain was not addressed in the document. No references were identified in the mission, vision, objectives, contextual analysis, implementation strategies, activities, outputs, or outcomes.

All included documents were coded by one analyst using a developed structured coding framework based on the food systems framework. To strengthen analytical process and coding consistency, two sequential rounds of coding were conducted independently by the same analyst. Discrepancies between coding rounds were systematically assessed, and where inconsistencies exceeded 20%, an additional reconciliation round would have been undertaken, though none of the policies exceeded this threshold. The setting of 20% discrepancy threshold was used pragmatically as an internal quality-control. This iterative process served as an intra-rater consistency and quality assurance procedure during content analysis.

## 3 Results

### 3.1 Overview

**Figure 1:**
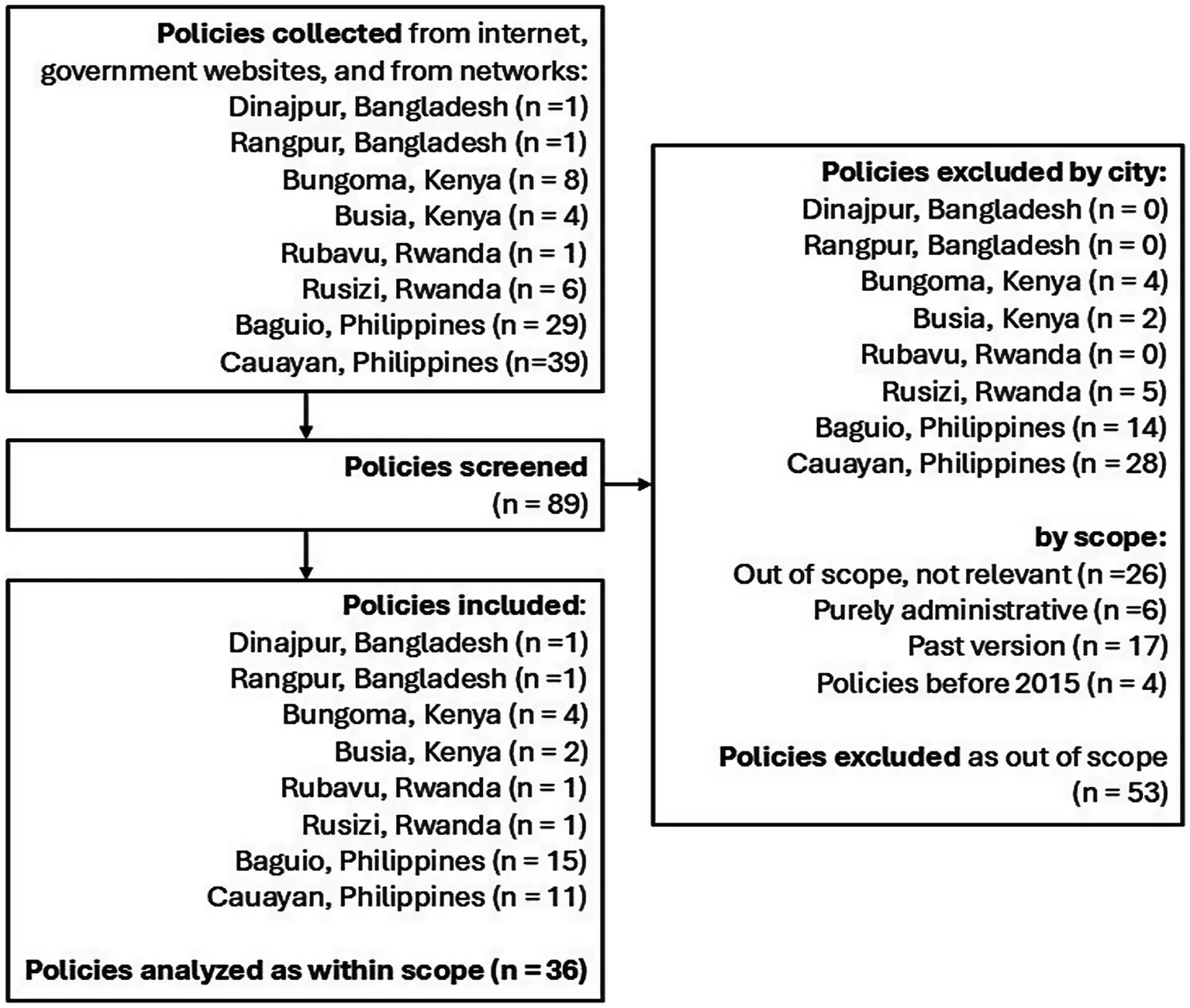
Policy mapping flowchart showing the collection of policies and action plans and subsequent screening process.

The systematic policy mapping process identified a total of 89 documents through formal and informal channels across the eight study cities (see Figure 1). Following a screening phase, 53 policies were excluded, primarily because they were out of scope—such as framework, strategies, commodity specific policies (*n* = 26), outdated versions (*n* = 17), were purely administrative (*n* = 6), or preceded the 2015 study period (*n* = 4). All 89 documents passed the screening for text readability, 87 documents are in English and two are in Bengali which has available English version. This resulted in a final analytical set of 36 policies within scope, with the highest concentration of included documents found in the Philippine cities of Baguio (*n* = 15) and Cauayan (*n* = 11), followed by Bungoma (*n* = 4) and Busia (*n* = 2) in Kenya, while the cities in Bangladesh and Rwanda each contributed one policy to the final analysis (*n* = 1 per city).

### 3.2 Characteristics of policies and action plans in terms of policy types

**Figure 2:**
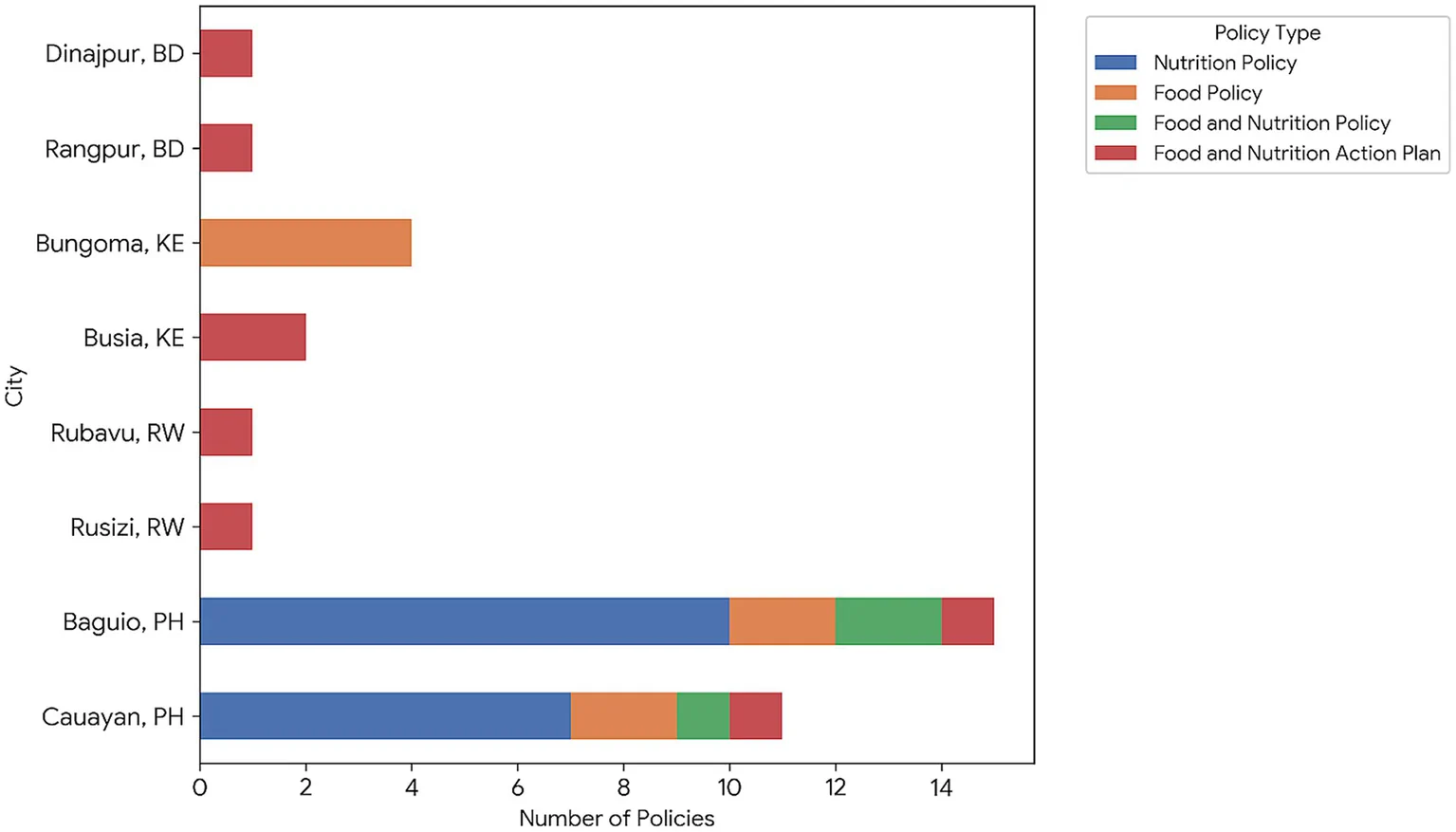
Distribution of policy types across the eight cities.

The distribution of the included policies (see Figure 2) reveals a concentration of Nutrition Policies in Baguio (*n* = 10) and Cauayan (*n* = 7), primarily consisting of local adoptions of national laws such as the Milk Code or the First 1,000 days Act. In contrast, the Kenyan city of Bungoma showed a focus on sectoral Food Policies related to agribusiness and soil management, while the cities in Bangladesh and Rwanda relied on single, comprehensive Food and Nutrition Action Plans to operationalize multi-sectoral nutrition goals. Only the Philippine cities featured the integrated Food and Nutrition Policy category, which explicitly links agricultural production to direct nutritional outcomes for vulnerable populations.

### 3.3 Policies and action plans according to thematic focus

**Figure 3:**
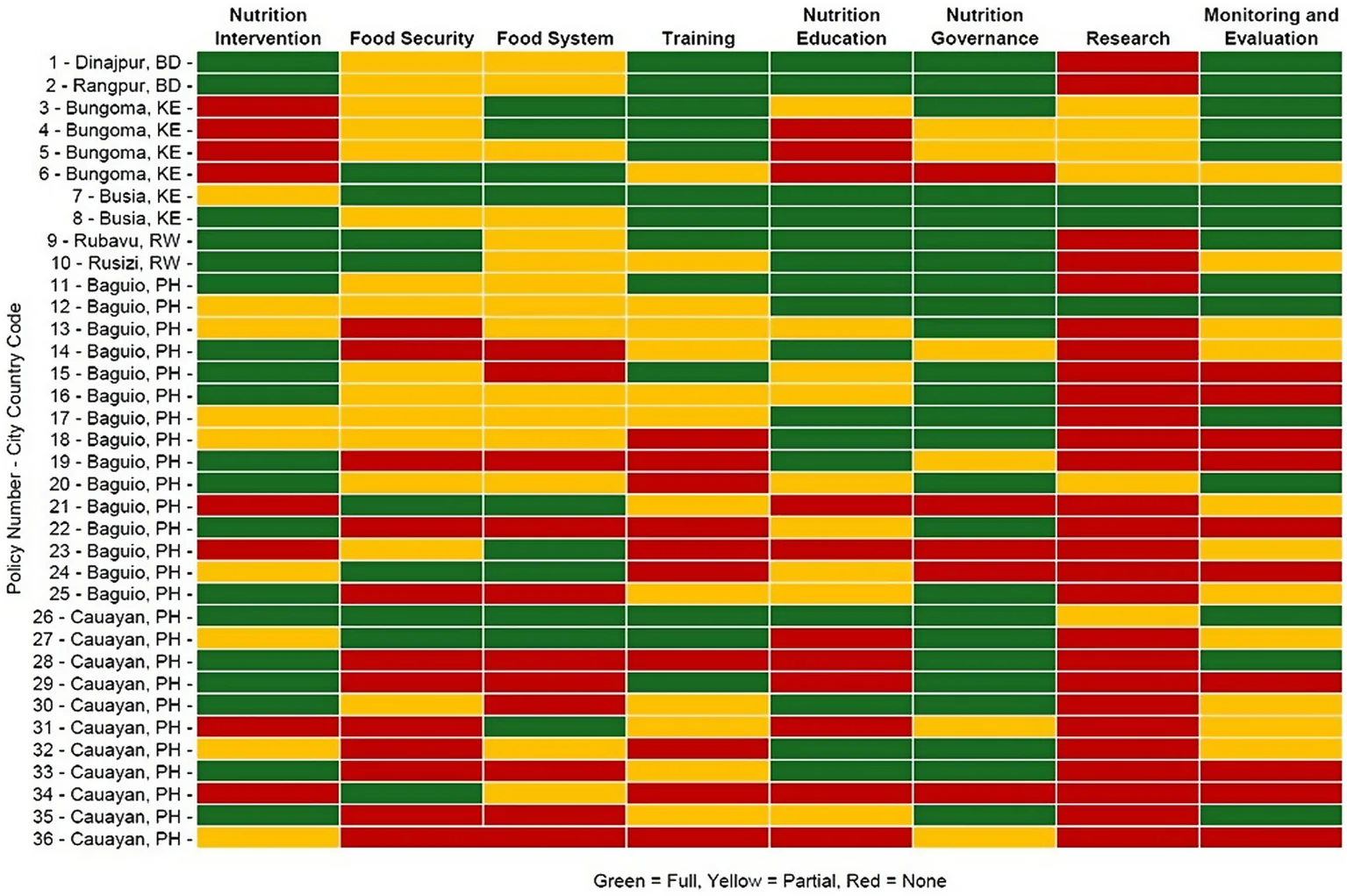
Distribution of policy focus across all collected policies and action plans across eight cities.

Across the 36 policy and action plan documents included in the analysis, Nutrition governance was a dominant thematic focus with 25 (69%) out of 36 were focused on this, particularly in the Philippines but also reflected in policies from Bangladesh, Kenya, and Rwanda (see Figure 3). These documents consisted of policies that outline structures and processes for coordinating, steering, and sustaining nutrition and food-system action within government. Nutrition intervention was the second most common theme, appearing in 53% of documents (19 total). These policies aimed to improve nutritional status by addressing nutrient deficiencies, diet quality, and nutrition-related disorders. Nutrition education was also seen in 44% or 16 of the documents, these policies included communication or behavior-change concerns to improve nutrition-related knowledge, attitudes, or practices. While monitoring and evaluation appeared as a component in 15 policies (42%), indicating less than half of policies incorporated indicators, reporting systems, and implementation tracking mechanisms. On the other hand, research or evidence generation was the least frequent thematic area. Only three policies (8%) emphasized the role of research, while 6 policies (17%) partially addressed this, and 27 policies (75%) did not refer to this. Food security and food systems thematic domains were commonly addressed partially, with 15 policies each (42%) in these domains, indicating that these themes were often acknowledged but not comprehensively laid out in the documents. Overall, the findings showed that the policy landscape across the study sites was strongly oriented toward governance and nutrition-specific interventions, while research or evidence generation, food systems integration, and broader food security dimensions remained comparatively less well-covered.

Across cities, differences in thematic priorities across were evident. The Nutrition Action Plans for Dinajpur and Rangpur in Bangladesh focused on nutrition intervention, training, education, governance, and monitoring and evaluation. As illustrated in Figure 3, these thematic areas were marked green in both cities, while research, food security, and food systems were either partially addressed or absent. In Kenya, Bungoma and Busia showed stronger focus toward food systems and capacity development compared with the other countries. In Bungoma, three out of the four policies demonstrated a food system’s focus, reflecting policy concerns on agro-business, food safety, value chains, and market systems. Similarly, training and monitoring and evaluation were strongly represented in three out of four Bungoma policies. Busia displayed a similar pattern, with attention to food system’s governance and institutional strengthening rather than direct nutrition interventions. In Rwanda, the two city level policies from Rubavu and Rusizi both focus on nutrition intervention, food security, nutrition education, and nutrition governance. However, neither policy demonstrated a strong food systems or research focus.

In the Philippines, which contributed the largest number of policies (*n* = 26), nutrition governance was the dominant theme reflected in 18 policies (69%). Nutrition intervention was also a focus in 14 policies (54%), while nutrition education appeared a focus in 10 policies (39%). However, food security and food system’s themes were less consistently being covered, with only five policies (19%) and six policies (23%), respectively, fully referring to these domains. The Philippine policy landscape therefore appeared heavily oriented toward governance and nutrition programming, with comparatively fewer policies addressing broader structural food systems transformation. Across all countries, research and evidence generation consistently remained thematic area that is left behind, indicating limited institutional importance on evidence generation and operational research within local food and nutrition policies.

### 3.4 Policies and action plans in terms of focus in the food system value chain

**Figure 4:**
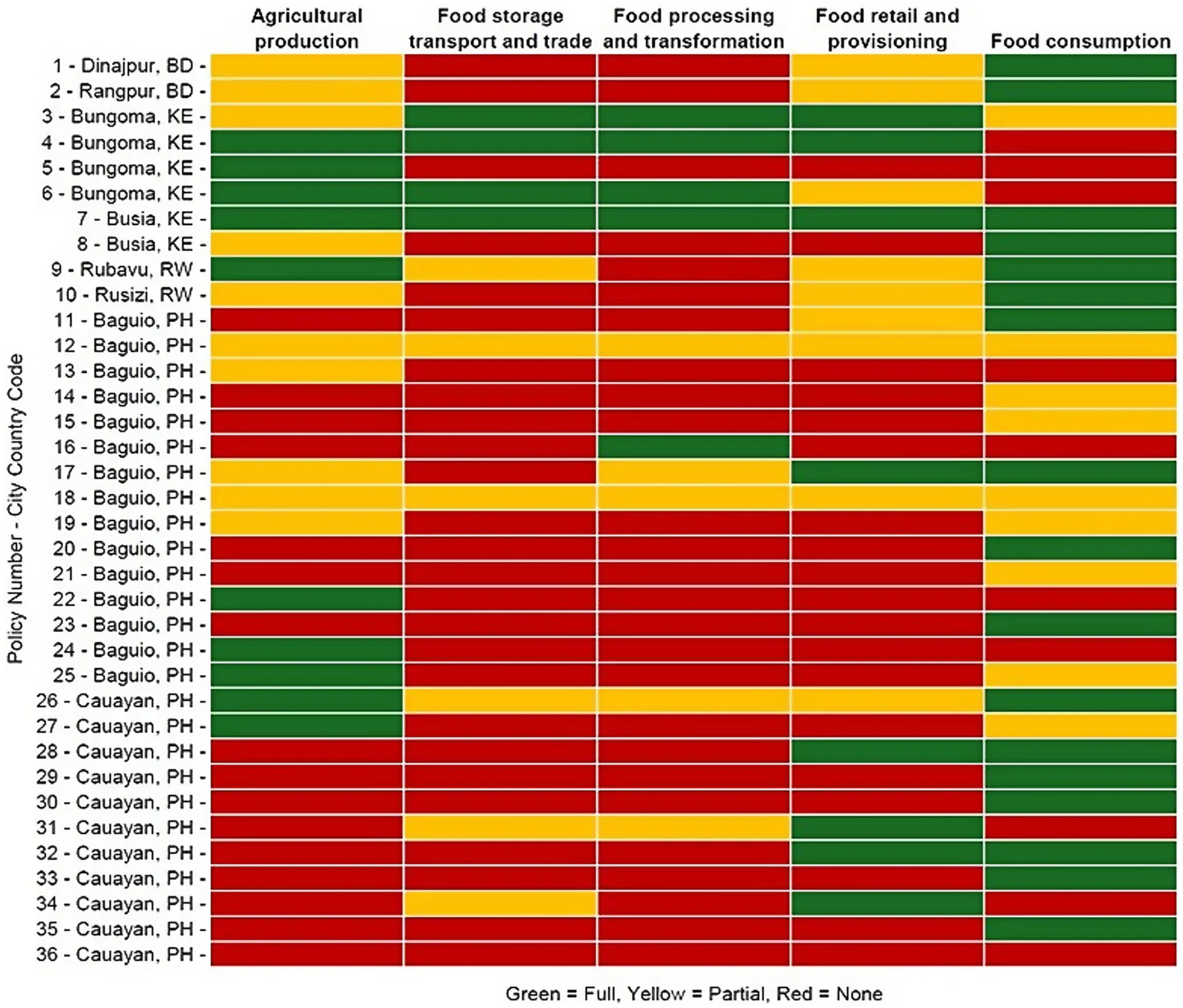
Heatmap of collected policies and action plans in terms of food system value chain.

The analysis of the 36 policy and action plans documents in terms of the food supply system value chain reveals a fragmented landscape, where interventions are heavily concentrated at the terminal ends of the chain while the mid-stream segments remain largely unaddressed (see Figure 4). Food consumption is the most integrated stage, with 47% (*n* = 17) of policies demonstrating a “Full” (green) focus and an additional 25% (*n* = 9) showing “Partial” (yellow) integration, primarily through infant feeding and dietary diversity programs. Similarly, Agricultural production receive some level of attention in 56% of documents (*n* = 20 combined green and yellow), often through the promotion of home gardening or agribusiness initiatives. However, a critical policy concern exists in the middle of the value chain; both Food storage, transport and trade and Food processing and transformation are entirely absent from 72% (*n* = 26) of the analyzed documents. This limited policy attention to mid-stream food system functions may have implications for food systems resilience in secondary cities, particularly in contexts experiencing rapid urbanization, climate-related disruptions, infrastructure limitations, and post-harvest losses affecting perishable and nutrient-dense foods.

Furthermore, over half of the policies (53%, *n* = 19) lack explicit measures for Food retail and provisioning, indicating that local governance is currently better equipped to address how food is grown and eaten than how it is safe-guarded, moved, or marketed. Similarly, few policies explicitly addressed regulatory instruments, incentives, taxation mechanisms, or operational standards related to sustainable food production, food transport systems, or resilient supply-chain management.

### 3.5 Policies and action plans in terms of addressing food security and nutrition outcomes

**Figure 5:**
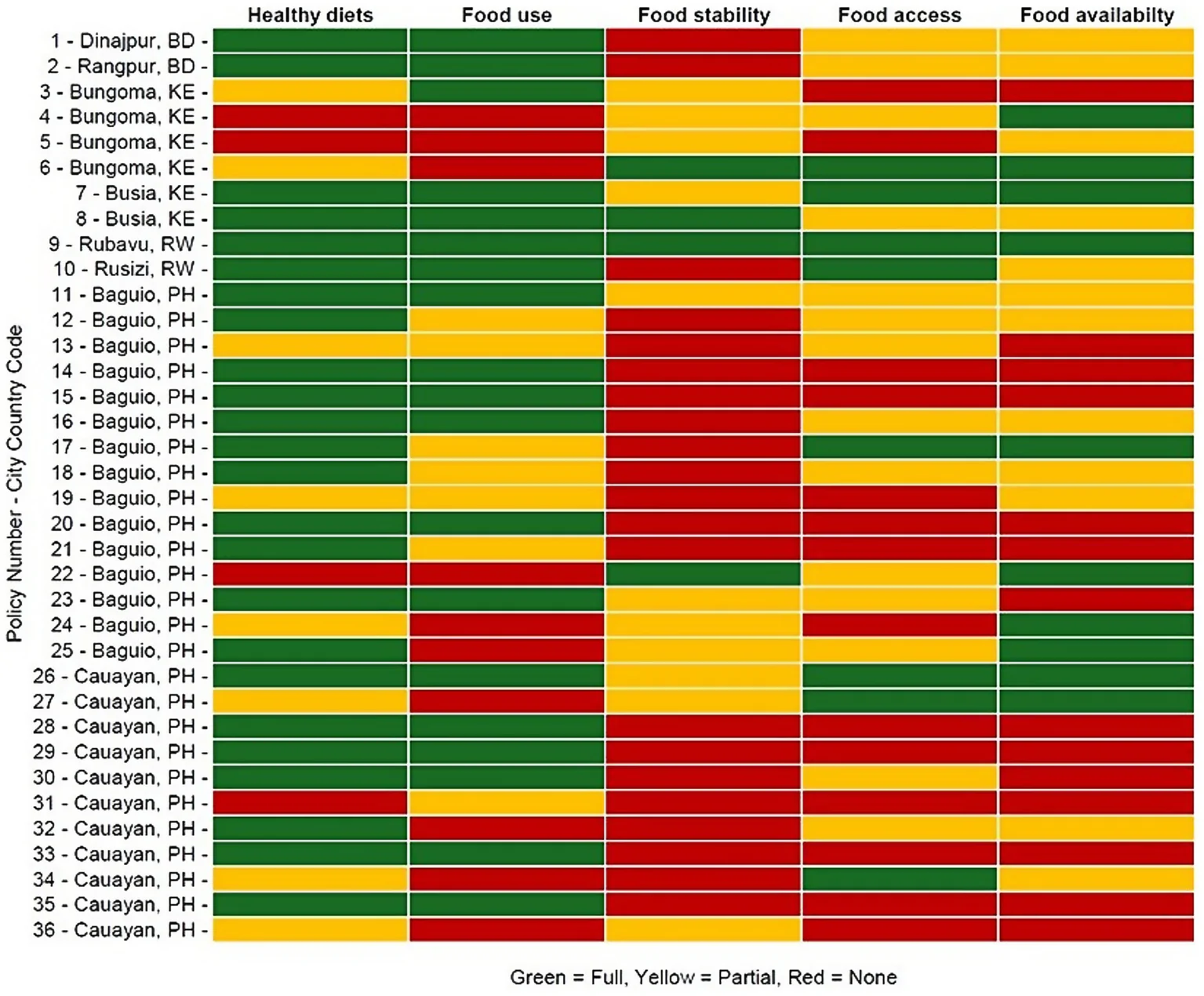
Heatmap of all collected policies and action plans in terms of addressing food security and nutrition outcomes.

The analysis reveals that while most of the policies and action plans are strongly oriented toward biological and individual level nutrition outcomes, they under-address the structural pillars of food security. Healthy diets emerged as the primary area of focus, with 67% (*n* = 24) of policies receiving a “Full” (green) rating, followed by Food use (hygiene and preparation), which is a major objective in 53% (*n* = 19) of the documents (see Figure 5). In contrast, structural dimensions of the food system are not well covered; Food stability is the least addressed outcome, with 58% (*n* = 21) of policies providing no explicit measures to manage shocks or seasonal volatility. Similarly, Food access (affordability and physical connectivity) and Food availability are entirely absent from 39% (*n* = 14 each) of the reviewed documents.

## 4 Discussion

This policy mapping across eight secondary cities in Bangladesh, Kenya, Rwanda and the Philippines shows that while nutrition is clearly written into formal policy documents, they are unevenly developed and partially aligned with a food systems approach. The 36 policies falling into the inclusion criteria (out of 89 retrieved documents), with a majority in Baguio and Cauayan and few documents in Dinajpur, Rangpur, Rubavu and Rusizi, mirror both differences in decentralization set up and in local policy-making capacity. In more devolved contexts such as Kenya and the Philippines, local governments are mandated to prepare and adopt sectoral or thematic plans (e.g., City/County Nutrition Action Plans, local ordinances for national laws), which may explain the volume of city level documents identified compared with the more unitary systems of Bangladesh and Rwanda, where guidance is within national plans and only selectively translated into standalone local policies (48, 49). This pattern is consistent with comparative work showing that decentralization has been associated with an increase number and variety of local nutrition and health policies, though not necessarily their coherence or implementation quality (50, 51).

The high number of Nutrition Policies in the Philippine cities, many of which are local enactments of national frameworks such as the Milk Code and the First 1,000 Days Act, highlights how statutory devolution under the Local Government Code appears to have contributed to a broad local policy landscape. This finding aligns with previous analyses of the Philippine Plan of Action for Nutrition, which document an active practice of local nutrition planning but point to variable alignment with broader food and agriculture concerns such as the food systems framework (52). In contrast, Bungoma’s focus on agribusiness and soil management policies and the reliance on a Food and Nutrition Action Plan in the Bangladesh and Rwanda sites reflect more sectoral or centrally managed approaches. Similar set up have been observed in other low- and middle-income countries (LMICs), where national multi-sectoral nutrition plans coexist with relatively narrow local sector strategies in agriculture or health which may be less responsive to broader determinants of undernutrition (53, 54).

Across cities, Nutrition Governance emerged as the most consistently and strongly addressed domain, with nearly 70% of policies providing clear coordination mechanisms and designated leadership coherent with the study of Amparo et al. (34) analyzing the food and nutrition security policies in the Philippines. This echoes the global trend, after the Scaling Up Nutrition (SUN) movement and adoption of the Global Nutrition Targets, toward formalized governance structures and inter-sectoral coordination platforms (51, 54). Our findings therefore contribute to the evidence that many countries have developed the structure of governance through committees, focal points, and coordination mechanisms, even in secondary cities and subnational settings (34, 39). However, the relative strength of governance provisions contrasts with the limited integration of evidence product, monitoring and outcome measurement which was absent or weak in most of the documents. This is consistent with previous reviews highlighting that, while national nutrition plans often calls for evidence-based policy, they rarely embed explicit commitments or budgets for monitoring, evaluation, and local research (23, 55, 56). In urban and peri-urban contexts in particular, the literature has underscored a shortage of locally generated data on food environments, dietary patterns and city wide inequities, which may constrain adaptive, area sensitive policy responses (17, 57). Our study provides concrete evidence of this research gap at city level in four LMICs.

The strong emphasis on direct Nutrition Interventions and Education such as maternal and child health services, infant and young child feeding (IYCF) counseling, and behavior change communication situates these secondary city policies within the nutrition specific paradigm described by Bhutta et al. (58), who showed that scaling up such interventions can substantially reduce child stunting and mortality. The focus on healthy diets and food use outcomes in the collected documents also resonates with linking dietary counseling, breastfeeding promotion and complementary feeding interventions to improved child growth and diet quality (59–61). This focus may reflect the continued importance of direct nutrition interventions in settings with high stunting prevalence in several study sites in Rubavu (35%), Cauayan (28.8%, Isabela Province) and Baguio (23.4%) where direct nutrition interventions remain important in addressing immediate determinants of undernutrition.

Our findings also show that domains related to Food Security and Food Systems are only partially covered, and that policy attention is biased toward the production and consumption as first and last segment of the food value chain while middle segments (storage, transport and trade; processing and transformation; retail and provisioning) are limited. This pattern may constrain the extent to which local policies engage food systems comprehensively in relation to nutrition outcomes, despite growing consensus that urban nutrition has increasingly been linked to food environments, value chains and market dynamics (37, 62, 63). While nutrition sensitive agriculture is increasingly recognized, most policies still focus on farm production and household consumption rather than on post-harvest handling (23, 64). Work in African and Asian secondary cities has documented similar gaps, especially around informal food retail, even though these are crucial for affordable diets among low-income urban households (65, 66).

These findings are also consistent with conceptual frameworks highlighting the relationship between nutrition outcomes and broader food security determinants (e.g., UNICEF (67); HLPE (37)) and with more recent calls to integrate stability and resilience into food system governance in the context of climate change, pandemics, and price shocks (68, 69). Evidence from the 2007 to 2008 food price crisis, the COVID-19 pandemic, and recurrent climate related disasters shows that neglecting stability, access and availability in policy design leaves urban populations, particularly low-income groups, highly vulnerable to disruptions, even when health and nutrition services are in place (70–72). Our results thus confirm for secondary cities what global analyses have observed at national level that there is a commitment to improving diets and child nutrition, but comparatively little policy attention to the systems and infrastructures that determine whether safe, nutritious food is reliably available and affordable (3, 18).

Beyond policy presence alone, the effectiveness of urban food systems also depends on implementation capacity, political leadership, financing, infrastructure, and broader socio-environmental conditions (2, 38, 39). Cities such as Rubavu and Rusizi in Rwanda, Baguio and Isabela Province where Cauayan City is located in the Philippines, combine high stunting prevalence with exposure to climatic and market shocks, including rainfall variability, cross-border trade disruptions, and disaster risk from flooding or landslides (73–76). Although the reviewed documents occasionally referred to resilience, emergency preparedness, or multi-sectoral coordination, explicit operational strategies related to transport systems, energy infrastructure, cold-chain development, urban land constraints for agriculture, and climate adaptation mechanisms were generally limited. In such contexts, the absence of explicit strategies for food stability (e.g., storage systems, contingency stocks, market monitoring, and social protection triggers) may undermine progress on chronic malnutrition, as seasonal and shock-related food insecurity persist (77, 78). Furthermore, relatively limited attention was observed regarding fiscal and regulatory instruments such as incentives for sustainable food production, taxation approaches, or market-based mechanisms to encourage healthier and environmentally sustainable food environments.

Overall, our mapping is coherent with existing literature on multi-sectoral nutrition and urban food systems in several respects. First, it confirms that most LMICs have now institutionalized nutrition governance structures and direct interventions, particularly around the first 1,000 days, breastfeeding and micronutrient supplementation (54, 58). Second, it supports evidence that multi-sectoral nutrition plans often remain health sector centered, with limited operationalization of food systems, social protection, and urban planning components at local levels (9, 23). Third, it parallels observations from urban food system studies showing that local governments frequently address agriculture, home gardening, or school feeding, but rarely regulate or invest in value chain infrastructure and food environments (37, 79).

To close persistent malnutrition gaps, policies must move beyond governance and direct interventions to integrate mid-stream value chains and structural food security determinants into local planning (62, 63). Evidence from this study is consistent with the need to integrate risk and resilience measures into local policies (19, 69). Furthermore, future investment could consider national support for city-level data systems to better enable local governments to track and adapt their interventions.

### 4.1 Strengths and limitations

A key strength of this study is its exhaustive and systematic, cross-country mapping of nutrition and food system related policies across eight secondary cities in four diverse LMIC contexts. By applying a common analytical framework to documents from Bangladesh, Kenya, Rwanda and the Philippines, the research allows for structured comparison of policy types, domains, value-chain coverage and nutrition and food security outcomes across different governance set up and nutritional burdens. The integration of a food system value chain lens and the disaggregation of food security into availability, access, use and stability dimensions go beyond many prior policy reviews that focus primarily on health sector or nutrition specific content.

However, several limitations must be acknowledged. First the presence or absence of policy provisions does not necessarily reflect implementation fidelity or concrete action dimensions such political leadership, funding allocation, or infrastructural development. Second, cities with relatively few but comprehensive documents may in practice be implementing some of activities under national programs which then may not be reflected in local policies and action plans. Third, the policy and action plan documents were coded and analyzed by one analyst rather than two independent coders. The absence of independent double coding may limit reproducibility and introduces the possibility of analyst bias in interpretation and categorization. To enhance coding consistency, the analyst conducted two independent rounds of coding using a predefined coding framework based on the food systems framework. This iterative approach was intended to improve intra-rater consistency and reduce subjective interpretation during coding. Fourth, there is unequal distribution of policy documents across countries, with the Philippine cities contributing a larger number of policies than the other study sites. This may have influenced the overall thematic patterns observed in the analysis. Fifth, contextual differences in governance, economic profile, and agro-ecological setting strongly vary that should be considered when interpreting cross-city comparisons. To address this, disaggregated country and city level analyses were conducted to support more context-specific interpretation of the findings. Finally, the study does not directly link policy content to measured nutrition outcomes over time; therefore, causality between policy characteristics and outcome measures such as stunting or diet quality cannot be inferred.

## 5 Conclusion

This multi-country policy mapping in secondary cities in Bangladesh, Kenya, Rwanda and the Philippines described the different policies and action plans in terms of their characteristics, types, thematic priority, food value chain focus and food security and nutrition outcomes they address. The study reveals that local policies remain only partially aligned with a comprehensive food systems approach. Gaps persist in mid-stream value chain functions such as storage, transport, processing and retail and in structural food security pillars such as availability, access and stability, especially in settings facing high stunting and exposure to climatic and market shocks. Furthermore, research, monitoring and evaluation through local evidence generation are lacking from city level policy texts, limiting adaptive, context specific responses.

To accelerate improvements in child nutrition and resilience in secondary cities, national and local authorities, together with initiatives like Nutrition in City Ecosystem project, should build on existing governance structures and nutrition specific platforms while deliberately expanding policy attention to food system infrastructures, markets and shock responsive mechanisms. Integrating research and monitoring within local policy frameworks will be essential to understand how urban food systems function in these settings and to design interventions that effectively bridge the gap between agricultural production, food environments and nutrition and health outcomes.

## Data Availability

The original contributions presented in the study are included in the article/Supplementary material, further inquiries can be directed to the corresponding author.
