# Supplementary material for: Are secondary cities in Asia and Africa ready for food systems transformation? Policy insights from Bangladesh, Kenya, Rwanda, and the Philippines
Source: Front Public Health. 2026 Aug 31;14:1852336. doi: 10.3389/fpubh.2026.1852336 (PMC13572720; doi:10.3389/fpubh.2026.1852336)
Supplement: Supplementary file 1 [file Data_Sheet_1.PDF]

Supplementary Material

Are Secondary Cities in Asia and Africa Ready for Food Systems Transformation? Policy Insights from Bangladesh, Kenya, Rwanda, and the Philippines

Daniel Gacutan Salunga[1,2], Kaspar Wyss[1,2]
[1]Swiss Tropical and Public Health Institute, Allschwil, Switzerland
[2] University of Basel, Basel, Switzerland

SECTION 1: Government types and nutrition and food systems policies and plans and its implementation in the four countries

TABLE 1 Overview of decentralisation and policy authority across countries and comparison of government types and nutrition and food systems policies and plans and its implementation

Country	Type of government	National policy authority (who sets the policies and plans?)	Implementation of policies and plans (centralized or decentralized?)
**Bangladesh**	Unitary parliamentary republic	Bangladesh National Nutrition Council (BNNC) being chaired by the Prime Minister, Ministry of Health and Family Welfare; National Nutrition Service.<br><br>National Plan of Action on Nutrition	Mixed: central policy & coordination; service delivery largely via decentralized health tiers (community clinics, upazila/district). (Central policy, decentralized delivery).
**Kenya**	Unitary presidential republic with constitutional devolution to counties	National Government (Ministry of Health) sets policy/standards (KNAP). (Family Health Kenya)<br><br>Kenya Nutrition Action Plan	Decentralized: counties implement through County Nutrition Action Plans; counties deliver health & agriculture functions.
**Rwanda**	Presidential republic (unitary)	Central government (Ministry of Health, Ministry of Agriculture and Livestock Development, national coordination bodies) develops policy.	Mixed / centrally steered: policy developed centrally; implementation is routed through districts/sector structures — policy emphasizes national + decentralized coordination.

		Rwanda National Food and Nutrition Policy	
**Philippines**	Unitary presidential constitutional republic, statutorily decentralized through RA 7160 (Local Government Code)	National Nutrition Council (NNC) / DOH produce PPAN and national guidance.<br><br>Philippine Plan of Action for Nutrition	Decentralized: implementation predominantly by LGUs (provinces/municipalities/barangays) with national oversight and technical support; PPAN is operationalized at LGU level.

NutriPoliCities Study

Nutrition and Food Systems policies and action plans
and food environment types and consumer perceptions
in Secondary Cities in Bangladesh, Kenya, Rwanda and the Philippines

POLICY REVIEW GUIDE I:

Coding Process for Nutrition and Food Systems Policies and Action Plans – Systematic Mapping and Content Analysis

Daniel Gacutan Salunga and Kaspar Wyss
Swiss Tropical and Public Health Institute, Allschwil, Switzerland
University of Basel, Basel, Switzerland

January 2025 – Allschwil, Switzerland

Purpose of the Policy Review Guide I - Codebook
This codebook provides standardized criteria for coding and classifying city-level policies related to nutrition and food systems. It ensures consistency in coding and supports transparent, replicable analysis. All coding decisions must follow the operational definitions and decision rules provided.

Unit of Analysis
The unit of analysis is a policy and action plan document
Drafts, enacted, or implemented versions are eligible if they meet inclusion criteria.

Inclusion & Exclusion Criteria

Inclusion
A document is included if it:
1. is issued by the city government or statutory bodies and is accessible, and readable
2. is drafted, enacted and implemented nutrition and food system policies and action plans
3. relates to nutrition, food systems, food security, health, agriculture, markets, food safety, or consumer environments
4. covers or affects the selected secondary cities
5. falls within the study period (January 2015 to January 2025, most recent version of the policy or plan was used)
6. It is in English or in local language – if in local language, it will be forwarded to research partners for translation however all policies and plans collected are in English

Exclusion
Exclude documents that:
1. are general development plans without any food or nutrition content
2. are private-sector documents
3. are academic research papers
4. have no relevance to food, nutrition, or food systems
5. are program, activity and administrative reports documents in nature
6. are past versions of the policy or plans

Coding Procedures
Step-by-Step Process
1. Read full policy document.
2. Apply decision tree to assign a single policy type.

3. Code each thematic area (nutrition, food system domain, governance, etc.).
4. Extract supporting quotes.
5. Enter all values into the coding sheet.

Quality measure
- Second round of analysis after reading all the policies

Table 2.1. Type of policy
Each policy type is mutually exclusive, meaning any document can fall into only one category. Adapted from the conceptual definitions from Dwyer (2016) (Reference Module in Food Science) and converted them into practical, document-classification criteria suitable for the study.

**Type of policy**	**Operational definition and exclusive criteria**	**CODING CRITERIA / CHECKLIST FOR EACH CATEGORY**
1. Nutrition Policy	A formal document issued by a government or authoritative local body whose primary and explicit purpose is to improve or protect nutritional status, dietary intake, or nutrition-related health outcomes of the population.<br>It includes clear intentions related to nutrition (e.g., reducing undernutrition, obesity, micronutrient deficiencies, promoting healthy diets).<br><br>Exclusive criteria:<br>A document is classified as a Nutrition Policy only if nutrition is the central focus and not just mentioned as one component within broader food or development strategies.	The document must:<br>✓ Explicitly aim to improve nutrition or diet-related health outcomes<br>✓ Include nutrition as the primary focus (not secondary or incidental)<br>✓ Contain measurable nutrition goals OR nutritional problem analysis<br>✓ Be strategic or regulatory, not operational<br><br>The document must NOT:<br>✗ Focus primarily on agriculture or food system functioning<br>✗ Be structured as an action plan (no activity lists, timelines)<br>✗ Only mention nutrition in passing
2. Food Policy	A policy document that focuses on food production, food supply, food safety, agriculture, trade, distribution, markets, or the general functioning of the food system,	The document must:<br>✓ Focus primarily on one or more food-system domains:<br>• production, supply chains, storage

	without explicitly foregrounding public health or nutrition objectives.<br>It may influence nutrition indirectly but does not position nutrition or health outcomes as a stated priority.<br><br>Exclusive criteria:<br>Classify a document as Food Policy only if it addresses food system issues without explicit goals to improve public health or nutritional status.	• markets and trade<br>• food prices, consumer protection<br>• food safety, quality<br>✓ Have no explicit nutrition or health improvement objective<br><br>The document must NOT:<br><br>✗ Include nutrition as a central policy goal<br>✗ Include nutrition targets, dietary guidelines, or health outcomes<br>✗ Present itself as a multisectoral food & nutrition policy
3. Food and Nutrition Policy	A formal policy that integrates both food-system–related goals (e.g., food security, markets, production, supply, retail environment) and explicit nutrition or public health goals (e.g., diet quality, nutrition status).<br>It is written to promote coordinated, multisectoral action on food and nutrition together.<br><br>Exclusive criteria:<br>A document qualifies as Food and Nutrition Policy only if it simultaneously:<br>1. addresses food system functions and<br>2. sets explicit nutrition or public health targets.<br>(If a document emphasizes only one domain—food or nutrition—it must be placed in the corresponding single-domain category instead.)	The document must:<br>✓ Integrate both food system and nutrition objectives<br>✓ Include explicit statements on how food system actions relate to nutrition outcomes<br>✓ Present a multisectoral coordination mechanism (health + agriculture + local government, etc.)<br><br>The document must NOT:<br>✗ Focus solely on nutrition or solely on food<br>✗ Be only an implementation plan (e.g., action plan)
4. Food and Nutrition Action Plan	An implementation-oriented document that outlines specific strategies, interventions, timelines, responsibilities, and resources for carrying out an existing food, nutrition, or food-and-nutrition policy.	The document must:<br>✓ Translate a policy (Food, Nutrition, or Food & Nutrition Policy) into operational steps<br>✓ Include:

	It operationalizes the policy through actionable steps.<br><br>Exclusive criteria:<br>A document is classified as an Action Plan only if it:<br>• details how a food, nutrition, or integrated policy will be implemented, AND<br>• presents concrete actions, activities, or implementation frameworks (e.g., budgets, performance indicators, timelines).<br>Policy documents without explicit action steps should not fall in this category.	• activities<br>• timelines<br>• implementing agencies<br>• budgets (if applicable)<br>• monitoring indicators<br>✓ Provide detailed implementation guidance for local or national actors<br><br>The document must NOT:<br><br>✗ Introduce entirely new policy directions<br>✗ Function primarily as a high-level policy<br>✗ Lack clear action steps

Table 2.1.1. Summary Table

**Category**	**Central Focus**	**Mentions Nutrition?**	**Mentions Food System?**	**Includes Action Steps?**	**When to Classify**
Nutrition Policy	Nutrition & health outcomes	Yes – core focus	Optional	No	When nutrition is the main purpose
Food Policy	Food system functioning	No (or incidental)	Yes – core focus	No	When food system focus lacks explicit nutrition aims
Food and Nutrition Policy	Integrated food + nutrition goals	Yes – explicit	Yes – explicit	No	When policy links food system strategies with nutritional outcomes
Food and Nutrition Action Plan	Implementation of policy	Yes / No (depends on related policy)	Yes / No	Yes – detailed actions	When a document operationalizes a policy through concrete strategies

Table 2.1.2. CODING SHEET TEMPLATE

**Variable**	**Description**	**Response Options**
Country	Study country	Bangladesh / Kenya / Rwanda / Philippines

City	Secondary city included in Objective 1	Dinajpur, Rangpur, Bungoma, Busia, Rubavu, Rusizi, Baguio, Cauayan
Document Title	Full official title of the policy/plan	Text
Document Year	Year of publication/approval	YYYY
Document Type	Policy, Plan, Ordinance, Strategy, Framework, etc.	Text

Table 2.1.3. Classification Using Decision Tree

**Variable**	**Description**	**Response Options**
Contains implementation actions?	Activities, timelines, indicators, budgets	Yes/No
Designed to operationalize a higher-level policy?	As per decision tree	Yes/No
Primary purpose is nutrition/health outcomes?	Yes/No	
Document integrates nutrition + food systems?	Yes/No	
Focuses mainly on food system domains?	Yes/No	
Final Classification Code	Category derived from decision tree	1 = Nutrition Policy<br>2 = Food Policy<br>3 = Food and Nutrition Policy<br>4 = Food and Nutrition Action Plan<br>5 = Out of Scope

Table 2.2. Assessment of policy focus in terms of thematic focus

Complete operational definition set for each type of policy thematic focus / domain, adapted from Adhikari et al. (Nutrition and Food Security in Nepal). 3-level rating criteria (Green–Yellow–Red) for coding, with precise operational rules that match policy mapping and content analysis framework.

**Type of Policy Thematic Focus / Domain**	**Operational definition**	**Criteria for coding**
Nutrition Intervention	Policies that directly aim to improve nutritional status through specific interventions that address nutrient deficiencies, diet quality, or nutrition-related disorders.	Green–Yellow–Red Criteria<br>Coder should review BOTH:

<table>
<tr>
<td></td>
<td>Includes:
Supplementation (e.g., vitamin A, iron, calcium)
Food fortification (e.g., iodized salt, fortified rice)
Treatment of undernutrition (SAM/MAM management)
Nutrition counseling targeting diet modification
Maternal, child, adolescent, or elderly nutrition interventions
Growth monitoring and promotion
Excludes:
Broad health services without nutrition-specific components
Agricultural interventions unless linked to nutrition outcomes</td>
<td rowspan="3">Mission / Vision / Goals / Objectives / Introduction / Context

Key Actions / Activity Lists / Outputs / Outcomes / Indicators

A color rating is assigned for each thematic domain.

GREEN — Full Presence
Operational Definition

The domain is a primary focus of the policy/plan.

Criteria (must meet at least 2 of 3):
✓ Mentioned explicitly and prominently in: Mission / vision / goals / objectives
AND/OR
✓ Highlighted in introductory or situational analysis
AND
✓ Reflected in key actions, outcomes, or operational activities

Meaning:
The domain is central to the intention and operationalization of the document.

Example
A plan whose main goal is “improving household food security” and has actions to</td>
</tr>
<tr>
<td>Food Security</td>
<td>Policies that address the FAO three pillars of food security:

Availability
Production, distribution, or supply of sufficient food.

Accessibility
Economic and physical access to food (affordability, safety nets, markets).

Utilization
Food safety, hygiene, and household-level use of food for adequate nutrition.

Excludes:
Food system activities not linked to availability/access/utilization.</td>
</tr>
<tr>
<td>Food System</td>
<td>Policies covering the entire chain of food system functions, from production to consumption and quality control.

Includes:
Agricultural production</td>
</tr>
</table>

	• Food storage and transport<br>• Trading and marketing<br>• Processing (small- or large-scale)<br><br>• Retail, provisioning, markets, food stalls<br>• Food safety and quality assurance systems<br><br>Excludes:<br>Purely nutrition-specific actions unless they relate to the food chain.	improve market access, storage, and food distribution.<br><br>**YELLOW** — Partial Presence<br>Operational Definition<br>The domain is addressed but not consistently or centrally.<br><br>Criteria (any 1 applies):<br>✓ Mentioned in mission/vision/objectives but not translated into actions<br>OR<br>✓ Appears only in actions, outputs, or activities but not mentioned in mission/vision/objectives<br>OR<br>✓ Addressed in the document but only as a secondary/supporting domain<br><br>Meaning:<br>The domain appears but is not strongly integrated into the policy logic.<br><br>**RED** — None<br>Operational Definition<br>No meaningful reference to the domain.<br><br>Criteria:<br>✗ Not mentioned in mission/vision/objectives/context<br>✗ Not reflected in key actions or outputs<br>✗ Not implied or referenced indirectly
Training	Policies aimed at improving the capacity and skills of human resources responsible for food, nutrition, agriculture, or health-related services.<br><br>Includes:<br>• Training for health workers, agricultural officers, food inspectors<br>• Capacity-building programs<br>• Workshops or upskilling on nutrition, food safety, food systems<br><br>Excludes:<br>Community nutrition education (classified separately under Nutrition Education).	
Nutrition Education	Any communication or behavior-change policy to improve nutrition-related knowledge, attitudes, or practices.<br><br>Includes:<br>• Information, education, and communication (IEC) materials<br>• Behavior Change Communication (BCC)<br>• Awareness campaigns<br>• Dietary guideline dissemination<br>• Community nutrition sessions<br>• Counseling related to food choices and healthy diets	

	Excludes:<br>Training of staff (classified under Training).	Meaning:<br>The document has zero engagement with the domain.
Nutrition Governance	Policies that outlines structures and processes for coordinating, steering, and sustaining nutrition and food-system action within government.<br><br>Includes:<br>• Multisectoral coordination committees<br>• Planning, budgeting, and accountability mechanisms<br>• Institutional capacity development<br>• Political commitment statements<br>• Roles and responsibilities for agencies or LGUs<br><br>Excludes:<br>Monitoring and evaluation (classified separately), though linked.	
Research	Policies that outline strategies to generate evidence to support food and nutrition decision-making.<br><br>Includes:<br>• Needs assessments<br>• Baseline/midline/endline research<br>• Operational research<br>• Studies on nutrition status, food systems, markets<br>• Evidence generation for policy development<br><br>Excludes:<br>Routine monitoring activities (classified under M&E).	
Monitoring and Evaluation	Activities related to tracking the implementation, outputs, outcomes, or impact of food or nutrition policies.<br><br>Includes:<br>• Monitoring indicators	

	• Evaluation frameworks<br>• Progress reviews<br>• Performance tracking systems<br>• Reporting mechanisms<br>• Surveillance systems<br><br>Excludes:<br>Research for knowledge generation (classified under Research).	

Table 2.2.1. Summary Table (Quick Reference for Coding)

Domain	Operational Definition	Green (Full)	Yellow (Partial)	Red (None)
Nutrition Intervention	Direct nutrition-specific actions	Central in goals + actions	Mentioned OR acted on, but not both	Not mentioned at all
Food Security	Availability, access, utilization	Central in goals + actions	Partial mention	None
Food System	Value chain activities	Core focus + actions	Mentioned but not central	None
Training	Capacity building for staff	Explicit goals + detailed training actions	Mentioned OR some minor activity	None
Nutrition Education	IEC/BCC for public	Featured in goals + actions	Mentioned OR minor action	None
Nutrition Governance	Coordination, institutional roles	Governance central + structured roles	Mentioned but weak	Not addressed
Research	Evidence generation	Research goal + action steps	Some research reference	None
Monitoring & Evaluation	Tracking implementation	Indicators + M&E framework	Mentioned only	None

Table 2.3. Assessment of policy focus in terms of Food Supply System - Value Chain Focus
Complete operational definition set for the Food Supply System – Value Chain Focus
Green / Yellow / Red scoring criteria, tailored specifically to each value chain domain.

Operational Definitions: Food Supply System – Value Chain Focus
These domains may overlap, and a policy or plan can score Green/Yellow/Red in each.
Each definition is adjusted to match a food systems framework relevant to local governments in secondary cities.

**Food Supply System - Value Chain Focus**<br>(can cut across)	**Operational definition**	**Criteria for coding**
Agricultural production	Policies or action plans addressing primary food production, including:<br><br>Includes:<br>• Crop cultivation, vegetable/fruit production<br>• Livestock rearing<br>• Poultry, fisheries, aquaculture<br>• Urban agriculture, rooftop/backyard gardening<br>• Input support (seeds, tools, fertilizer, irrigation)<br>• Climate-resilient agriculture, agroecology<br>• Extension services aimed at improving production<br><br>Excludes:<br><br>• Processing, storage, or market activities (coded under other domains)<br>• Nutrition messaging without production action<br>• Environmental or land-use plans unrelated to food production	Green–Yellow–Red Criteria (Specific to Each Value Chain Domain)<br><br>These criteria allow coder to classify the extent to which the policy addresses each value chain stage.<br><br>The scoring must be applied independently for each domain.<br><br>GREEN — Full Presence (Primary Focus)<br>A domain scores Green if BOTH criteria are met:<br>1. Strategic Level<br>The domain is explicitly stated in the policy's:<br>• Mission<br>• Vision<br>• Goals<br>• Objectives<br>Introductory/context statements<br>AND<br><br>2. Operational Level<br>The domain is directly reflected in:<br>• Key actions<br>• Activities<br>• Outputs<br>• Implementation strategies<br>• Outcomes<br><br>Meaning:
Food storage transport and trade	Policies or plans addressing post-harvest handling, logistics, and market-related trade.<br><br>Includes:<br>• Post-harvest storage<br>• Cold-chain systems<br>• Transport and distribution logistics<br>• Wholesale market systems<br>• Food import/export rules (if applicable)	

	• Market linkages, aggregation centers<br>• Supply chain continuity / food flow in emergencies<br><br>Excludes:<br>• Retail activities (coded separately)<br>• Food safety inspections (unless tied to transport/storage conditions)	The policy or plan treats this domain as a central pillar or priority area.<br><br>**YELLOW** — Partial Presence (Secondary or Implied Focus)<br>A domain scores Yellow if EITHER criterion is met (but not both):<br>1. Mentioned only in strategy-level text<br><br>Mentioned in:<br>mission/vision/objectives/context<br>BUT NOT represented in actions.<br><br>OR<br><br>2. Present only in operational actions<br><br>The domain appears in:<br>• activities<br>• outputs<br>• outcomes<br>BUT NOT mentioned in mission/vision/objectives/context.<br><br>OR<br><br>3. Addressed as supportive or minor content<br>Referenced in the plan but not central.<br><br>Meaning:<br>The domain is recognized but not strongly integrated into the overall policy logic.
Food processing and transformation	Policies that facilitate or regulate the transformation of raw agricultural products into processed or semi-processed foods.<br><br>Includes:<br>• Small/medium-scale food processing<br>• Milling, drying, smoking, fermenting, packaging<br>• Value addition programs (women's groups, SMEs)<br>• Food industry regulation at local level<br>• Equipment support or training for processing<br>• Food safety requirements tied to processing facilities<br><br>Excludes:<br>• Distribution or retail actions<br>• Nutrition-specific food fortification (coded under Nutrition Interventions)	
Food retail and provisioning	Addresses how food becomes available to consumers through retail or provisioning systems.<br><br>Includes:<br>• Wet/open markets<br>• Street food vendors	

	• Hawkers, kiosks, temporary stalls<br>• Supermarkets, groceries, sari-sari stores, dukas<br>• Market infrastructure improvements (shade, sanitation, drainage)<br>• Licensing, standards, food vendor regulation<br>• Public food provisioning (food banks, feeding centers if food distribution oriented)<br><br>Excludes:<br>• Household consumption practices<br>• Production-to-market transport (coded under Storage/Transport/Trade)	**RED** — None (Absent)<br>A domain scores Red if:<br>• Not mentioned in mission, vision, goals, objectives, context<br>• AND<br>• No actions/outcomes/strategies relate to the domain<br>• AND<br>• No implicit references exist<br><br>Meaning:<br>The policy or plan does not address this part of the food value chain.
Food consumption	Policies related to how households/individuals obtain, prepare, and consume food.<br><br>Includes:<br>• Consumption patterns<br>• Dietary intake practices<br>• Household food utilization<br>• Meal frequency, dietary diversity<br>• Food safety at point of consumption<br>• Consumer behavior, meal preparation practices<br><br>• Interventions influencing household consumption (unless clearly nutrition-specific, which is coded separately)<br><br>Excludes:<br>• IEC/BCC nutrition education (coded under Nutrition Education)<br>• Retail or market access actions	

Table 2.3.1. Summary Table (Quick Reference for Coding)
Food Supply System – Value Chain Focus

Value Chain Domain	Operational Definition	Green (Full Presence)	Yellow (Partial Presence)	Red (None)
**1. Agricultural Production**	Policies or plans addressing primary food production, including crops, livestock, fisheries, aquaculture, and urban agriculture. Includes inputs (seeds, tools, fertilizer), agroecology, and extension services.	Domain is explicitly stated in mission/vision/goals/context AND actions include production activities (urban agri, rearing, fisheries, input support).	Mentioned in mission/vision/objectives but not in actions; OR present in actions (e.g., seed distribution, gardening) but not in mission/vision/objectives.	No mention of agricultural production, inputs, extension, or urban agriculture.
**2. Food Storage, Transport & Trade**	Policies addressing post-harvest handling, storage, cold-chain, logistics, wholesale systems, and market linkages. Includes supply chain continuity during disruptions.	Explicit strategic framing on storage/logistics/trade AND concrete actions (e.g., building storage facilities, improving transport flows, wholesale markets).	Mentioned in strategy but no actions OR actions exist (e.g., storage facility, supply distribution) but not in strategic framing.	No reference to storage, logistics, food trade, transport, or supply chain continuity.
**3. Food Processing & Transformation**	Policies regulating or supporting transformation of raw agricultural products into processed foods (milling, drying, smoking, fermenting, packaging). Includes value addition programs and food industry regulation.	Processing/value addition is a stated goal or objective AND actions support processing facilities, SMEs, training, or regulation.	Mentioned in strategy but not in actions OR appears only in actions (e.g., equipment support) but not in strategic text.	No reference to processing, transformation, food industry regulation, value addition, or related actions.

**4. Food Retail & Provisioning**	Policies addressing how food reaches consumers through wet markets, street vending, stalls, shops, supermarkets, and provisioning systems (food banks, canteens). Includes market infrastructure, regulation, licensing, sanitation.	Retail/provisioning appears in mission/vision/goals/context AND actions directly target markets, vendors, retail infrastructure, or provisioning systems.	Mentioned in strategy but not reflected in actions OR retail/provisioning actions exist (market upgrades, vendor training) but not in strategic text.	No mention of food retail, markets, vendors, provisioning systems, or market infrastructure.
**5. Food Consumption**	Policies addressing how people obtain, prepare, and consume food, including dietary habits, meal patterns, home food safety, and household utilization practices.	Consumption practices or household utilization are stated in strategic text AND appear in actions (cooking practices, household safety, consumption patterns).	Mentioned only in mission/vision/objectives OR appears only as actions (dietary practices, home food hygiene) with no strategic framing.	No references to food consumption, household preparation, utilization, dietary practices, or related behaviors (excluding nutrition education).

Notes for Coding

- Each domain is scored independently (not mutually exclusive).
- Policies may score Green in multiple domains.
- The table is designed for city-level food system policy mapping in Bangladesh, Kenya, Rwanda, and the Philippines.
- Coding should record verbatim evidence from the policy to justify each rating.

Table 2.4. Assessment of policy focus in terms of what is the major outcomes it would like to address in terms of Food security and nutrition outcomes

These outcomes reflect the intended results a policy or plan aims to improve in a secondary city's food security and nutrition situation.
A policy can score multiple domains.

<table>
<tr><th>Food security and nutrition outcomes</th><th>Operational definition</th><th>Criteria for coding</th></tr>
<tr><td>Healthy diets</td><td>A policy or plan is focused on healthy diets when it aims to ensure that individuals and households consume foods and beverages that:
• provide adequate nutrients
• prevent malnutrition (undernutrition and overweight/obesity)
• prevent micronutrient deficiencies
• reduce diet-related NCD risks
• promote balanced, diverse, safe diets

Evidence may include references to:
• Dietary diversity
• Balanced meals
• Reducing junk/ultra-processed food intake
• Fruit/vegetable consumption
• Breastfeeding and complementary feeding quality
• Reducing salt, sugar, fat intake</td><td rowspan="2">GREEN–YELLOW–RED ASSESSMENT CRITERIA (SPECIFIC TO EACH OUTCOME)
These criteria determine how strongly a policy or plan targets each outcome.

A domain can score Green, Yellow, or Red, independently.

GREEN — FULL (Primary Focus)

The policy/plan is classified as Green for a domain when:

1. Strategic Level Evidence (must be present)
The outcome appears in:
• Mission
• Vision
• Goals
• Objectives
• Introductory/context statements
• Problem analysis
AND

2. Operational Level Evidence (must also be present)
The outcome appears in:
• Key actions
• Strategies
• Activities
• Outputs</td></tr>
<tr><td>Food use</td><td>A policy is focused on food use when it addresses household- and individual-level practices that affect how food contributes to health, including:
• Food preparation, storage, cooking
• Safe handling of food
• Household-level food hygiene
• Nutrient bioavailability (e.g., soaking, fermenting, fortification use)
• Meal preparation skills
• Food safety behaviors at home
This domain links food intake with nutrition outcomes through proper utilization.</td></tr>
</table>

<table>
<tr>
<td>Food stability</td>
<td>A policy is focused on food stability when it aims to reduce risks of food interruptions over time, due to:
Seasonal variability
Supply chain disruptions
Shocks (climate events, price volatility, pandemics, disasters)
Market fluctuations
Political or economic instability
City-level examples include:
Emergency food supply planning
Disaster risk reduction (DRR) related to food
Price stabilization or anti-hoarding measures
Strengthening buffer stocks</td>
<td rowspan="3">Outcomes
Interpretation:
The domain is a central, intentional outcome of the policy or plan.

YELLOW — PARTIAL (Secondary, Indirect, or Limited Focus)
A domain is classified as Yellow if:
Either:
It appears in goals/objectives/context, but not in actions
OR
It appears in actions, but not in goals/objectives
OR
It is mentioned but plays a supporting or minor role
OR
It appears indirectly without explicit prioritization
Interpretation:
The domain is acknowledged but not central.

RED — NONE (Absent)
A domain is classified as Red when:

1. It does NOT appear in strategic text,

AND

2. It does NOT appear in actions/outcomes,

AND</td>
</tr>
<tr>
<td>Food access</td>
<td>A policy is focused on food access when it ensures residents can obtain adequate, safe, nutritious food, considering:
Physical access (distance, proximity, transport networks, walkability)
Economic access (prices, affordability, income constraints)
Social access (cultural acceptability, safety, non-discrimination)
Urban examples:
Market infrastructure development
Food subsidies or vouchers
Regulating food prices
Improving public transport to markets
Strengthening informal food vending livelihoods</td>
</tr>
<tr>
<td>Food availability</td>
<td>A policy is focused on food availability when it seeks to ensure that sufficient quantities of quality, safe, culturally appropriate food are consistently present in:</td>
</tr>
</table>

	• Markets<br>• Retail outlets<br>• Local supply chains<br>• Municipal food stocks<br>• Community food distribution systems<br><br>Urban examples:<br>• Strengthening urban agriculture<br>• Encouraging local food supply chains<br>• Ensuring supply continuity to public markets<br>• Slaughterhouse or fish landing site infrastructure	3. No implicit reference exists.<br><br>Interpretation:<br>The policy or plan does not address this outcome.

Table 2.4.1. Summary Table (Quick Reference for Coding)
Food Security & Nutrition Outcomes

Outcome Domain	Operational Definition	Green (Full Presence)	Yellow (Partial Presence)	Red (None)
**1. Healthy Diets**	Policies aiming to ensure individuals and households consume nutrient-adequate, diverse, balanced diets, preventing undernutrition, micronutrient deficiencies, and diet-related NCDs. Includes dietary diversity, fruit/vegetable intake, reduced intake of unhealthy foods, breastfeeding & complementary feeding quality.	Healthy diets are explicitly stated in mission/vision/goals/context AND actions highlight diet quality (e.g., complementary feeding quality, fruit/vegetable promotion, reducing unhealthy food intake).	Mentioned in strategic text but absent in actions OR appears only in actions (e.g., dietary counselling) but not in mission/objectives OR appears only as minor supportive content.	No mention of diet quality, dietary diversity, healthy eating practices, or improved dietary intake.
**2. Food Use (Utilization)**	Policies addressing how households prepare, store,	Utilization issues appear in mission/objectives/context AND	Mentioned in strategy but not translated into actions	No reference to food preparation,

	handle, and consume food safely and nutritiously. Includes household food hygiene, cooking practices, nutrient bioavailability, safe preparation, and storage behaviors.	actions include improved cooking practices, household hygiene, safe food handling, IYCF feeding practices.	OR actions include utilization (e.g., home hygiene training) but not in mission/objectives.	storage, household food hygiene, safe handling, or consumption practices.
**3. Food Stability**	Policies reducing risks of food supply interruptions due to seasonal variation, shocks, disasters, supply chain disruptions, price volatility, or conflict. Includes DRR, emergency food planning, buffer stocks, and price stabilization measures.	Stability or resilience is a stated objective AND actions include emergency food supply planning, buffer stocks, price stabilization, DRR, or supply continuity mechanisms.	Referenced in strategy but not in actions OR actions imply stability (e.g., building storage facilities) but not mentioned in mission/objectives.	No mention of shocks, seasonality, supply continuity, disaster-related food issues, or stability mechanisms.
**4. Food Access**	Policies ensuring residents have physical, economic, and social access to adequate, safe, nutritious food. Includes affordability, availability of markets, transport to food retail points, safety, and cultural acceptability.	Food access appears in mission/objectives/context AND actions include upgrading markets, improving transport to markets, price regulation, support to informal vendors, subsidies, affordability measures.	Mentioned in mission/objectives but no actions OR actions exist (e.g., market upgrades, vulnerable population support) but not stated in mission/context.	No mention of affordability, market access, transport to food points, barriers to access, or mechanisms improving access.
**5. Food Availability**	Policies ensuring sufficient quantities of culturally acceptable, safe, nutritious food are reliably present in markets, retail outlets, and local supply chains. Includes strengthening urban agriculture, supply continuity, distribution systems, and retail infrastructure.	Availability is included in mission/objectives/context AND actions include urban agriculture, distribution systems, local food production support, slaughterhouse/fish landing infrastructure, or market supply continuity.	Mentioned in strategic text but no actions OR actions supporting availability exist (e.g., urban agriculture, food storage) but not mentioned strategically.	No reference to food supply levels, market stock, local production to ensure availability, or distribution continuity.

Notes for Coding

- Score each outcome domain independently (documents can score Green, Yellow, or Red in multiple domains).
- Coding should provide verbatim evidence (quotes or page references) for transparency.
- The table is optimized for analysis of urban and secondary-city policies in BD, KE, RW, PH.

SECTION 3: Policies and Action Plan Documents

Table 3. List of Policies and Action Plan Documents

Policy Code	Country	City	Year / Period	Title of Policies / Action Plans
1	Bangladesh	Dinajpur	2023 to 2025	Dinajpur Municipality Multisectoral Nutrition Coordination Committee
2	Bangladesh	Rangpur	2023 to 2025	Rangpur City Multisectoral Nutrition Coordination Committee
3	Kenya	Bungoma	2023	Bungoma County Food Safety Policy 2023
4	Kenya	Bungoma	2023	Bungoma County Agribusiness Policy 2023
5	Kenya	Bungoma	2023	Bungoma County Agricultural Soil Management Policy 2023
6	Kenya	Bungoma	2023	Bungoma County Agriculture Sector Policy 2023
7	Kenya	Busia	2024 to 2029	Busia County Agri-Nutrition Implementation Strategy 2024 to 2029
8	Kenya	Busia	2023 to 2028	Busia County Nutrition Action Plan 2023 to 2029
9	Rwanda	Rubavu	2024 to 2025	Rubavu District Plan to Eliminate Malnutrition 2024 to 2025
10	Rwanda	Rusizi	2023 to 2024	Rusizi District Plan to Eliminate Malnutrition 2023 to 2024
11	Philippines	Baguio	2023 to 2025	Baguio City Nutrition Action Plan 2023 to 2025

12	Philippines	Baguio	2023	Baguio city Executive Order No. 82 Series of 2023 Amending Executive Order No. 152 S 2022 Entitled Reconstituting and Revitalizing the Baguio City Nutrtion Office and Redefining their Function
13	Philippines	Baguio	2023	Baguio City Executive Order No. 50, Series of 2023 Designating a City Nutrition Action Officer (CNAO), City Nutrition Program Coordinator (CNPC) and District Nutrition Program Coordinators (DNPC) to Enhance the Implementation of the City Nutrition Program and Barangay Nutrition Scholar Program
14	Philippines	Baguio	2023	Baguio City Resolution No. 478, Series of 2023 Enjoining All Barangay Nutrition Committees to Organize and Ensure Functionality of Breastfeeding Support Groups in All Barangays in the City of Baguio
15	Philippines	Baguio	2022	Baguio city Executive Order 51 series 2023 Creating a Sub-Committee to be Called the F1K First 1000 Days in Support of Republic Act No. 10028 Expanded Breastfeeding Promotion Act Republic Act 11148 Kalusugan at Nutrisyon ng Mag-Nanay Act and Executive Order No. 51 S. 1986 Milk Code
16	Philippines	Baguio	2022	Baguio City Resolution No. 274 S.2022 “Adopting the Provisions and Principles of Republic Act No. 11148 otherwise known as the “Kalusugan at Nutrisyon ng Mag-Nanay Act,” in the City of Baguio for the City Government of Baguio to Issue and Implement Effective Health Promotion Policies, Programs and Activities”
17	Philippines	Baguio	2022	Baguio City Resolution No. 273 S.2022 “Adopting the Provisions and Principles of Republic Act No. 11037 otherwise known as the “Masustansyang Pagkain Para sa Batang Pilipino Act,” in the City of Baguio for the City Government of Baguio to Issue and Implement Effective Health Promotion Policies, Programs and Activities”
18	Philippines	Baguio	2022	Baguio City Executive Order No. 152 series of 2022 “Reconstituting and Revitalizing the Baguio City Nutrition Committee (BCNC) Repositioning the City Nutrition Office and Redefining Their Functions Thereat
19	Philippines	Baguio	2022	Baguio City Resolution No. 178, Series of 2022 Designating and Including the Barangay Nutrition Scholars (BNS) Under the Coverage of Resolution No. 102, Series of 2022 as Barangay-Level Health Education and Promotion Officers (HEPOs) in the City of Baguio
20	Philippines	Baguio	2019	Baguio City Resolution no. 512 s.2019 “Adopting the IYCF Global targets for 2025 and RA no. 10028 (An Act Expanding the promotion of breastfeeding amending for the purpose of RA 7600, Otherwise known as “An Act providing incentives to all government and private health organizations with rooming-in and breastfeeding practices and for other purposes) in the City of Baguio”

21	Philippines	Baguio	2019	Baguio City Resolution No. 301 s.2019 “Adopting the Philippine Plan of Action for Nutrition 2017-2022 by the National Nutrition Council in the City of Baguio”
22	Philippines	Baguio	2019	Baguio City Resolution No. 143, Series of 2019 Confirming the Deed of Donation Made and Executed by the City Government of Baguio in Favor of the Department of Education–Baguio City Division for the Implementation of the “Gulayan sa Paaralan” Program in the Elementary and Secondary Schools in the City of Baguio
23	Philippines	Baguio	2018	Baguio City Ordinance No. 22 s. 2018: “Establishment of Lactation or Breastfeeding Stations in the Workplaces of the City Government and Public Offices, Business Establishments, Malls and Other Family-Oriented Businesses in the City of Baguio”
24	Philippines	Baguio	2018	Baguio City Resolution No. 236, Series of 2018 Requesting the Schools Division of Baguio City, Department of Education–Cordillera Administrative Region (DepEd–CAR) to Monitor the Strict Implementation of DepEd Order No. 13, Series of 2017 on Healthy Food and Beverage Choices in Schools and in DepEd Offices, and to Encourage Food Stores Within the Proximity of Schools to Adopt the Said DepEd Order
25	Philippines	Baguio	2015	Baguio City Resolution No. 198, Series of 2015 Strongly Endorsing Organic Backyard Gardening to Augment the Nutritional Needs of the Elderly and the Family
26	Philippines	Cauayan	2023 to 2025	Cauayan City Nutrition Action Plan 2023 to 2025
27	Philippines	Cauayan	2024	Cauayan City Ordinance No. 2024 560 An ordinance Requiring the Establishment of Communal Vegetable and Medicinal Gardens in Barangay Halls, Schools and Universities in the City of Cauayan Isabela and Providing Funds Thereof
28	Philippines	Cauayan	2024	Cauayan City Resolution No. 2024 060 Resolution Adopting the Executive Order No. 51 Otherwise Known as The Philippine Milk Code and Strengthening the Implementation and Acceptance through the Creation of the City Milk Monitoring Team in the City of Cauayan, Isabela
29	Philippines	Cauayan	2024	Cauayan City Resolution No. 2024 089 Resolution Adopting the Implementation of Republic Act No. 11148 Otherwise Known as "An Act Scaling up the National and Local Health and Nutrition Programs through a Strengthened Integrated Strategy for Maternal Neonatal, Child Health and Nutrition in the First One Thousand (1000) Days of Life, Appropriating Funds Therefore and for other purposes
30	Philippines	Cauayan	2022	Cauayan City Ordinance No. 2022 472 An Ordinance Creating the Children’s Welfare Code of Cauayan City, Isabela and Appropriating Funds Thereof

31	Philippines	Cauayan	2018	Cauayan City ORDINANCE NO. 2018 167 An Ordinance Regulating the Sanitation Standard rating for Food and Drinking Establishments in the City of Cauayan Isabela
32	Philippines	Cauayan	2018	Cauayan City RESOLUTION NO. 2018 039 Resolution Adopting the Department of Education Order No. 13 Series of 2017 Issued on March 14, 2017 "Policy and Guidelines on Healthy Food and Beverage Choices in Schools and in DepEd offices"
33	Philippines	Cauayan	2016	Cauayan City RESOLUTION NO. 2016 115 A Resolution to Ensure the Promotion of Good Nutrition in the First 1000 days in a Childs Life of National Nutrition Council Region 02
34	Philippines	Cauayan	2016	Cauayan City Resolution No. 2016 136 Resolution Creating Cauayan City Food Bank, that will form part of the Social Services Program of the LGU to help uplift the needs of Marginalized Sector of Society in the City of Cauayan, Isabela
35	Philippines	Cauayan	2015	Cauayan City EXECUTIVE ORDER NO. 01 SERIES OF 2015: Organization of the City Infant and Young Child Feeding Team
36	Philippines	Cauayan	2015	Cauayan City Resolution no. 2015 072 Resolution Adopting the National Policy on Nutrition Management in Emergencies and Disasters in the City of Cauayan, Isabela
